# Mid-term results in patients having tricuspidization of the quadricuspid aortic valve

**DOI:** 10.1186/1749-8090-9-29

**Published:** 2014-02-08

**Authors:** Meong Gun Song, Hyun Suk Yang, Dong Hyup Lee, Je Kyoun Shin, Hyun Keun Chee, Jun Seok Kim

**Affiliations:** 1 Department of Thoracic and Cardiovascular Surgery, Konkuk University Medical Center, Konkuk University School of Medicine, Seoul, South Korea; 2 Department of Internal Medicine, Konkuk University Medical Center, Konkuk University School of Medicine, Seoul, South Korea; 3 Department of Thoracic and Cardiovascular Surgery, College of Medicine, Yeungnam University, 317-1, Daemyungdong, Namgu, Daegu 705-035, South Korea

**Keywords:** Aortic valve, Quadricuspid, Reconstructive surgical procedure, Outcome

## Abstract

**Background:**

Quadricuspid aortic valve (QAV) is a rare congenital anomaly. We investigate the mid-term results of aortic valve reconstruction by tricuspidization in patients with QAV.

**Methods:**

We analyzed the outcome of eight consecutive patients who underwent aortic valve reconstruction surgery (AVRS) with pericardial leaflets with symptomatic quadricuspid aortic valve (QAV) disease between December 2007 and May 2012. AVRS consists of leaflet reconstruction and fixation of the sino-tubular junction in order to maintain coaptation of the new valve.

**Results:**

Six males and two females were included; ages ranged from 19 to 63 years (mean age, 51 years). According to Hurwitz and Roberts’s classification, three patients had type A, three patients had type B, one patient had type C, and one patient had type E. All patients had significant aortic regurgitation (AR): moderate in three patients, moderate to severe in one patient, and severe in four patients. Concomitant ascending aorta wrapping with an artificial vascular graft was performed in one case. There was no occurrence of mortality during the follow-up period (42.4 ± 18.0 months). No redo-operation was required. The NYHA functional class showed improvement from 2.1 ± 0.2 to 1.1 ± 0.2 (*p* = 0.008). The latest echocardiograms showed AR absent or trivial in seven patients, and mild in one patient. The aortic valve orifice area index (AVAI) was 1.03 ± 0.49 cm^2^/m^2^. Compared with preoperative echocardiograms, the left ventricular (LV) ejection fraction showed improvement from 57.6 ± 17.0 to 63.7 ± 13.2% (*p* = 0.036); the end-diastolic and end-systolic LV dimensions showed a significant decrease, from 63.5 ± 9.6 to 49.5 ± 3.1 mm (*p* = 0.012) and 43.6 ± 11.8 to 32.1 ± 5.4 mm (*p* = 0.012), respectively.

**Conclusion:**

In patients with QAV, AVRS with tricuspidization showed satisfactory early and mid-term results. Long-term follow-up will be necessary in order to study the durability of AVRS; however, it can be considered as a potential standard procedure.

## Background

QAV is rare, with an estimated prevalence of 0.013% to 0.043% [1]. Among 225 patients who underwent aortic valve surgery for pure aortic regurgitation (AR), two patients were found to have QAV (an incidence of 0.88%) [2]. Yotsumoto et al. [3] reported an incidence of 1.46%. Abnormal function of the quadricuspid aortic valve is a frequent occurrence; the most common abnormality is valvular insufficiency. Therefore, surgical correction is required as previous present aortic valve replacement or a few cases of aortic valve repair. We report on our experience with the mid-term outcome and review previously reported cases from the literature.

## Methods

We retrospectively analyzed eight patients who underwent aortic valve reconstruction surgery (AVRS) for treatment of QAV disease at Konkuk University Medical Center between December 2007 and May 2012 (Table 1). Informed consent was obtained from all patients, and the study was approved by the Institutional Review Board of Konkuk University. Six males and two females were included; ages ranged from 19 to 63 years (mean age, 51 years). According to Hurwitz and Roberts’s classification, three patients had type A, three patients had type B, one patient had type C, and one patient had type E. All of the patients had significant aortic regurgitation: moderate in three patients, moderate to severe in one patient, and severe in four patients. Concomitant ascending aorta wrapping with an artificial vascular graft was performed in one case and resection of fibrous subaortic membrane in one case.

**Table 1:** Baseline characteristics of patients

**Patient**	**Age**	**Gender**	**Hurwitz and Roberts type**	**Ascending aorta dimension (mm)**	**NYHA classification**	**AR grade**	**Associated anomaly**
1	48	M	E	41.2	II	Severe	
2	53	M	B	33.5	II	Severe	
3	63	M	C	43.2	II-III	Moderate to severe	
4	19	M	A	30.0	II	Moderate	
5	63	F	B	37.1	II	Severe	Fibrous subaortic stenosis
6	46	M	B	28.0	II	Moderate	
7	60	M	A	45.0	II	Severe	
8	55	F	A	38.5	II	Moderate	

Gender (M: male, F: female); NYHA: New York Heart Association; AR: aortic regurgitation.

Statistical analysis of the data was performed using SPSS 18.0 (IBM, Armonk, NY, USA). Categorical variables were expressed as number (proportions, %), and continuous variables were expressed as mean ± standard deviation. We used the Wilcoxon Signed Ranks Test as a non-parametric method. The value which is statistically significant is p value < 0.05.

### Surgical techniques

We performed AVRS using previously described techniques [4,5] for treatment of patients with QAV disease. Surgery was performed through a median sternotomy under moderately hypothermic cardiopulmonary bypass with antegrade and retrograde cold blood cardioplegia. The aortic valve was exposed and excised through a horizontal aortotomy 1.0 cm above the right coronary artery ostium.

**Figure 1:**
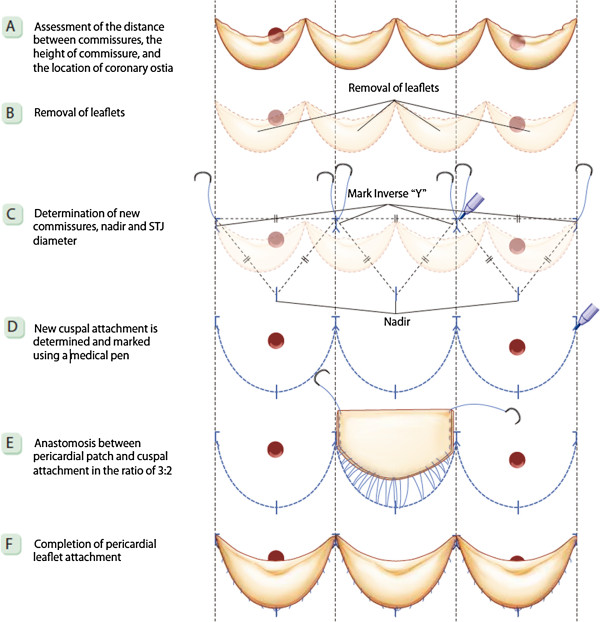
**Tricuspidization of a quadricuspid aortic valve. A, B, C, D, E, F** (permission by Song MG).

**Figure 2:**
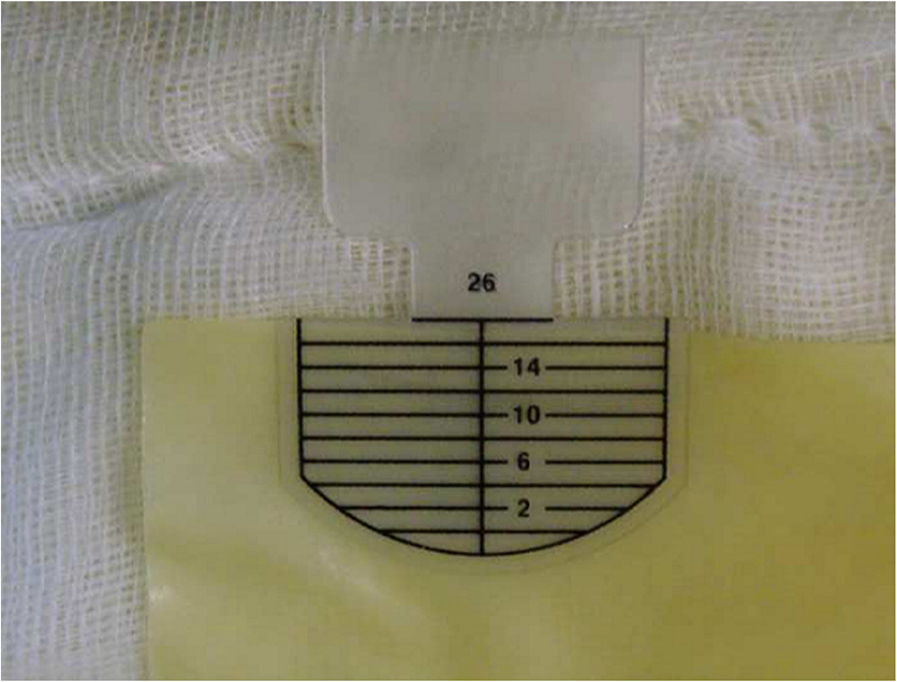
A 26-mm C-Leafcon® (ScienCity; Seoul, ROK) template among several sizes.

**Figure 3:**
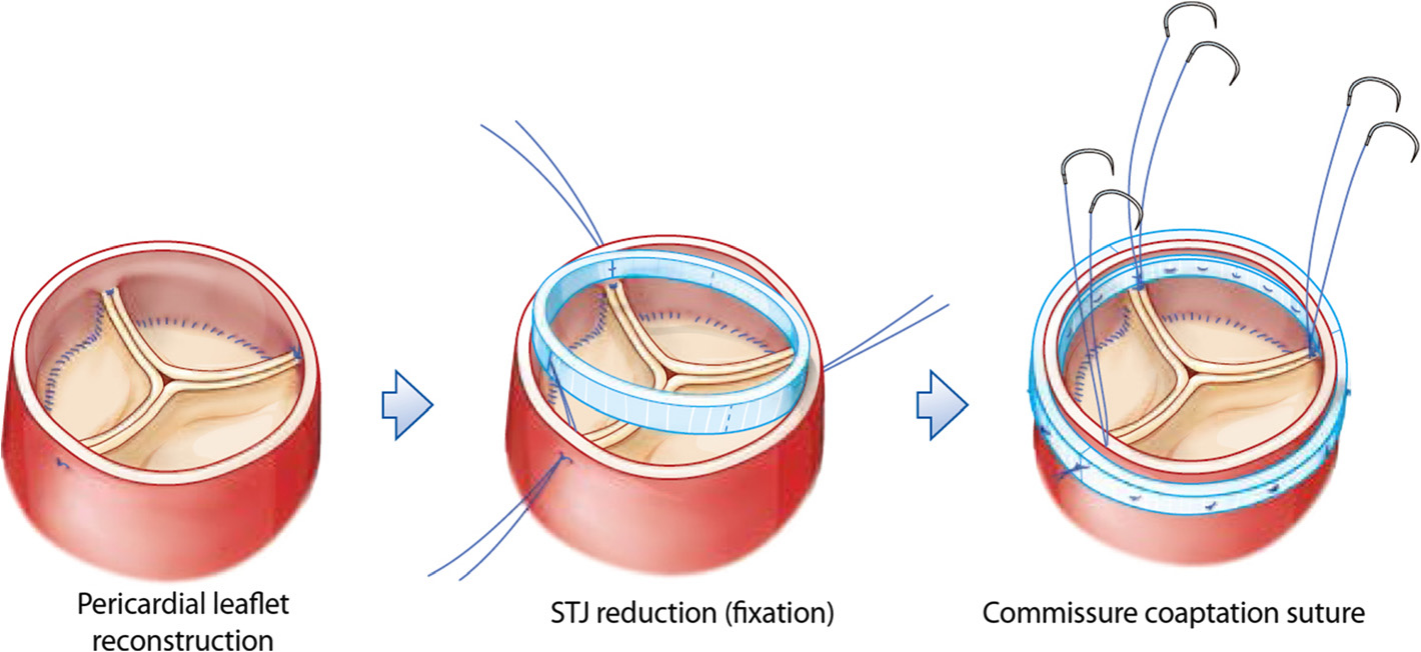
**Serial process of the pericardial leaflet reconstruction.** The sinotubular junction reduction, and the commissure coaptation suture (permission by Song MG).

For AVRS, the diameter of the new sinotubular junction (STJ) was first determined. Tricuspidization would appear to be most applicable to any type of QAV (Figure 1). In order to create new aortic leaflets, bovine pericardium (Supple Peri-Guard®, Synovis®, St Paul, MN, USA) was tailored to the leaflet size that would fit the STJ diameter. To simplify the construction of the pericardial leaflets, we used five templates of different sizes at 2.0-mm intervals. Each leaflet size was matched to an STJ diameter, which was also the size of the inner ring for STJ fixation (Figure 2). Subsequently, the new STJ was placed at 2.0 mm above the new commissures (at least 5.0 mm above both coronary ostia) and fixed together with a non-expandable inner ring and a softer outer ring or strip (ScienCity Co. Republic of Korea) using 18 interrupted 4–0 polypropylene mattress sutures. Coaptation sutures were made with a figure-eight 5–0 polypropylene suture at the upper leaflet margin 1–2 mm from each commissural end of the leaflets (Figure 3). The outer ring or strip for STJ fixation was 4–6 mm larger than the inner ring according to the thickness of the aortic wall. The size of the leaflet template was always the same as that of the inner STJ ring in the individual. The aortotomy was subsequently closed in the conventional manner.

## Results

Pre- and postoperative transthoracic echocardiographic studies were routinely performed before surgery, at discharge from the hospital, and then annually if there were no problems. Using the most recent echocardiographic data (28 ± 17 months), we examined the function of the repaired aortic valve and left ventricle: degree of aortic regurgitation, the aortic valve area index, left ventricular ejection fraction (LVEF), LV end-systolic dimension (LVESD), and LV end-diastolic dimension (LVEDD). There was no occurrence of mortality during the follow-up period (42.4 ± 18.0 months). No redo-operation was required. The NYHA functional class showed improvement from 2.1 ± 0.2 to 1.1 ± 0.2 (p = 0.008). The latest echocardiograms showed absent or trivial AR in seven patients, and mild in one patient. The AVAI were 1.73 ± 0.32 cm^2^/m^2^ and 1.03 ± 0.49 cm^2^/m^2^, respectively. Compared with preoperative echocardiograms, the LVEF showed improvement from 57.6 ± 17.0 to 63.7 ± 13.2% (p = 0.036); the LVEDD and LVESD showed a significant decrease, from 63.5 ± 9.6 to 49.5 ± 3.1 mm (p = 0.012) and 43.6 ± 11.8 to 32.1 ± 5.4 mm (p = 0.012), respectively (Table 2).

**Table 2:** Follow up echocardiographic data

	**Preoperative**	**Postoperative**	**P value**
LVEF (%)	57.6 ± 17.0	63.7 ± 13.2	0.036
LVESD (mm)	43.6 ± 11.8	32.1 ± 5.4	0.012
LVEDD (mm)	63.5 ± 9.6	49.5 ± 3.1	0.012
AVAI (CE, cm^2^/m^2^)	1.73 ± 0.32	1.03 ± 0.49	0.025

Data are given as mean ± standard deviation. Evaluated with Wilcoxon Signed Ranks Test.

LVEF; left ventricular ejection fraction; LVESD; left ventricle end systolic dimension; LVEDD; left ventricle end diastolic dimension; AVAI; aortic valve area index, CE; continuity equation.

## Discussion

QAV is rare; the incidence varies with diagnostic modality. Echocardiography reports range from 0.013% to 0.043%, which is likely an underestimate [1]. The incidence of coronary and cardiac anomalies associated with quadricuspid valve has been reported as approximately 18.3%, with coronary anomalies being the most common (10.2%) [6]. There were no coronary artery anomalies in the present series. We had one case of subaortic fibromscular stenosis.

In 1973, Hurwitz and Roberts described seven different morphologies of QAV (types A-G) based on leaflet size and the degree of cusp equality. The most frequent leaflet morphology types were A and B, both of which were most frequently regurgitant. Type A (four equal cusps; three cases), type B (three large equal cusps, one smaller cusp; three cases), type C (two larger equal cusps, two smaller equal cusps; one case), E (three equal cusps, one large cusp; one case) None of the patients in our group were type D (one large cusp, two intermediate cusps, one small cusp) type F (two large equal cusps, two smaller unequal cusps), or type G (four unequal cusps) [7]. The most common abnormality of QAV is valvular insufficiency. The predominant clinical findings and management issues in QAV relate to progressive AR with aging due to progressive leaflet fibrosis and progressive failure of leaflet coaptation. The quadricuspid aortic valve is replaced in the majority of patients requiring surgery; only a few cases of in situ surgical repair have been reported [8-10]. Schmidt et al. [11] reported surgical plasty repair via tricuspidization in two cases with two normal sized leaflets and two smaller leaflets using a combination of leaflet fusion, resection of the interposed commissure, and, in one patient, patch augmentation. AVRS in QAV may be more complex than that of the native tricuspid aortic valve. However, the procedure was simplified on the basis of the aortic root anatomy [12]. Fixation of the sino-tubular junction is very important new conception to maintain coaptation of the reconstructed valves [13]. The process showed previous figure presentation. Tricuspidization would appear to be most applicable to any type of QAV (Figure 1). Adult patients with QAV in our series did not have an enlarged ascending aorta (size 33–45 mm, mean 37 mm). Concomitant ascending aorta wrapping with an artificial vascular graft was performed in one case with dilated ascending aorta in size 45 mm and removal of fibrous subaortic membrane was performed in one case. There was no occurrence of mortality during the follow-up period (42.4 ± 18.0 months). No redo-operation was required. Hemodynamic results were satisfactory, as follows. The NYHA functional class showed improvement. The latest echocardiograms showed AR absent or trivial AR in seven patients, and mild in one patient. The AVAI were 1.73 ± 0.32 cm^2^/m^2^ and 1.03 ± 0.49 cm^2^/m^2^, respectively.

Compared with preoperative echocardiograms, the LVEF showed improvement, and the LVEDD and LVESD showed a significant decrease. Long-term follow-up will be necessary in order to assess the durability of repair procedures and material involving the quadricuspid valve. AVRS in patients with QAV disease can accomplish accurate coaptation and low valve gradient without turbulent flow; we expect favorable long-term durability of the new pericardial leaflets.

## Conclusions

The early and midterm results of QAV patients undergoing AVRS were satisfactory; however, they should be continuously followed for analysis of long-term results. Aortic valve reconstructive surgery as the tricuspidization of QAV can be considered as a potential standardized procedure.

## Abbreviations

QAV: Quadricuspid aortic valve; AVRS: Aortic valve reconstruction surgery; STJ: Sinotubular junction; AR: Aortic regurgitation; AVAI: Aortic valve area index; LVEF: Left ventricular ejection fraction; LVESD: Left ventricular end-systolic dimension; LVEDD: Left ventricular end-diastolic dimension; NYHA: New York Heart Association; CE: Continuity equation.

## Competing interests

The authors declare that they have no competing interest.

## Authors’ contributions

MS carried out patient recruitment, data collection, drafted the manuscript and provided advice. HY carried out patient recruitment, data collection, reviewed the statistics and draft the manuscript. DL initiated and designed the study, collected and analyzed the data and wrote the manuscript. JS, HC, and JK planned and carried out the surgical parts of the procedure. All authors read and approved the final manuscript.
